# Feature Selection and Predictors of Falls with Foot Force Sensors Using KNN-Based Algorithms

**DOI:** 10.3390/s151129393

**Published:** 2015-11-20

**Authors:** Shengyun Liang, Yunkun Ning, Huiqi Li, Lei Wang, Zhanyong Mei, Yingnan Ma, Guoru Zhao

**Affiliations:** 1 Shenzhen Key Laboratory for Low-cost Healthcare, and Shenzhen Institutes of Advanced Technology, Chinese Academy of Sciences, 1068 Xueyuan Road, Shenzhen 518055, China; E-Mails: sy.liang@siat.ac.cn (S.L.); yk.ning@siat.ac.cn (Y.N.); lihuiqi@siat.ac.cn (H.L.); wang.lei@siat.ac.cn (L.W.); 2 Chengdu University of Technology, No.1, Third East Road, Erxianqiao, Chengdu 610059, China; E-Mail: meizhanyong2014@cdut.edu.cn; 3 Beijing Research Center of Urban System Engineering, Beijing 100035, China; 4 College of mathematics and statistics, Shenzhen University, Shenzhen 518055, China

**Keywords:** feature selection, fall prediction, lower limb extremity, gait and balance, ground reaction force, sample entropy, KNN-based classifier

## Abstract

The aging process may lead to the degradation of lower extremity function in the elderly population, which can restrict their daily quality of life and gradually increase the fall risk. We aimed to determine whether objective measures of physical function could predict subsequent falls. Ground reaction force (GRF) data, which was quantified by sample entropy, was collected by foot force sensors. Thirty eight subjects (23 fallers and 15 non-fallers) participated in functional movement tests, including walking and sit-to-stand (STS). A feature selection algorithm was used to select relevant features to classify the elderly into two groups: at risk and not at risk of falling down, for three KNN-based classifiers: local mean-based k-nearest neighbor (LMKNN), pseudo nearest neighbor (PNN), local mean pseudo nearest neighbor (LMPNN) classification. We compared classification performances, and achieved the best results with LMPNN, with sensitivity, specificity and accuracy all 100%. Moreover, a subset of GRFs was significantly different between the two groups via Wilcoxon rank sum test, which is compatible with the classification results. This method could potentially be used by non-experts to monitor balance and the risk of falling down in the elderly population.

## 1. Introduction

A fall is defined as an event which results in a person coming to rest inadvertently on the ground or floor or other lower level, with or without loss of consciousness or injury. Falls have become the second leading health problem of unintentional injury deaths all over the world [1]. In recent years, more and more people have been paying attention to falls among the aging population. Several studies have been done to identify risk factors for falls. Generally, previous falls, gait and balance deficit were considered as important risk factors [2]. There are several fall risk assessment scales focused on elderly people. Perell *et al.* [3] summarized the most common fall risk assessment scales based on 21 articles published from 1984 to 2000. Among them, there were fourteen institution-focused nursing assessment scales, and six functional assessment scales, but the fall risk assessment chosen might vary depending on setting. In a community setting, the number of diseases and medications has no influence within short time periods, but mobility and balance play important roles in the prediction of falls [4].

The fall risk of community-dwelling is evaluated by functional balance tests, such as Timed Up and Go (TUG) test [5], Dynamic Gait Index (DGI) [6], Berg Balance Scale (BBS) [7], Tinetti Performance Oriented Mobility Assessment (Tinetti POMA) [8]. These tests are easy to quantify by a physician with clinical scores and they facilitate statistical processing. However, different versions of these tests can make comparisons difficult, such as the TUG which has reported threshold values that vary from 10 to 33 s in different literatures [9]. Therefore, an objective and simple test for predicting fall risk is very necessary. The article proposes an objective method to identify fall status predictors related to gait and balance pattern and based on ground reaction force (GRF) data collected by a force platform.

The foot pressure, which is measured by foot force sensing technology, plays a crucial role in gait and balance analysis. Platform systems are one foot force sensing technology, which can measure static and dynamic pressure [10]. Force platform measurements have been used as predictors of falls among elderly populations based on their functional movements, such as walking, standing and sitting. The force platform can provide valuable information regarding the vertical and horizontal components of the ground reaction force [11]. Ground reaction force is the foot pressure which acts on the body as a response to its weight and inertia during the contact of the human lower extremities with the supporting surface. It is widely used and researched by many scientists in areas such as discrimination between normal and abnormal gait [12], subject recognition [13], elderly fall prevention [14] and fall risk assessment [15].

In this article, a new and objective predictor of falls is proposed. Firstly, physical features are extracted from walking and balance tests of each subject and quantified by sample entropy. Then, each feature is separately trained and tested using KNN-based classifiers. The classification performances are estimated by a leave-one-out validation technique to compute overall accuracy, sensitivity, and specificity. Finally, the features are selected based on the highest accuracy, sensitivity, and specificity. The proposed method provides a way to analyze the gait and balance based on objective information and classify elderly people into fallers (persons at risk for falling ) and non-fallers (persons not at risk). This is the first step in the design of a fall risk assessment system that could be useful in evaluating balance and the risk of falling down.

The paper is organized as follows: Section 2 describes the experimental design and the way to acquire the data. Section 3 briefly presents feature extraction based on sample entropy. In Section 4 and Section 5, we present the outline of three KNN-based classification algorithms for selecting features and statistical analysis. The experimental results and discussion are presented in Section 6. Section 7 gives the conclusions.

## 2. Experimental Data Acquisition

### 2.1. Participants

In our experiment, a convenience sample of 38 participants over 65 years old, living in the community, was selected. Among them there were 21 females and 17 males, with an age range of 65–84 years, and a weight range of 40–90 kg. None of them had any neurologic or orthopedic condition that would affect their gait pattern. Participants were asked the following question: “During the past year, did you have a fall?” with two responses (yes/no). Those participants who reported a fall, were also asked to report the number of falls, not including falls resulting from unavoidable environmental hazards such as a chair collapsing or walking on ice which may affect balance. In a word, all participants were categorized as fallers or non-fallers, according to self-reported experience of at least one fall within the past year.

### 2.2. Force Platform Measurements

**Figure 1:**
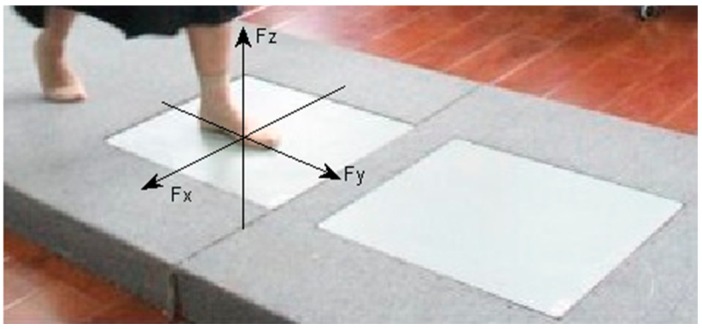
GRF components: F_x_ F_y_, and F_z_ on the multi-axis force platform. F_x_ F_y_, and F_z_ represent medial-lateral, anterior-posterior and superior-inferior GRF for foot during walking, respectively.

The tests in the study are functional compound movements to assess lower limb mobility, including walking, standing and sit-to-stand tests. The experimental procedures are described as follows:

The first record is the 3 m walking test. The subjects performed clear steps on two commercial force platforms (AMTI model OR6-7, Watertown, MA, USA) while walking at their normal and comfortable speed. They stopped at the end of the 3 m, turned back and did it again. Figure 1 shows a subject participating in such an experimental setting. Multi-axis force platforms were used to collect ground reaction force data. The data consist of three components F_x_ , F_y_ and F_z_, where F_z_ reflects the vertical forces of the mass; F_x_, F_y_ correspond to the horizontal friction that forces the subject along the horizontal plane.

**Figure 2:**
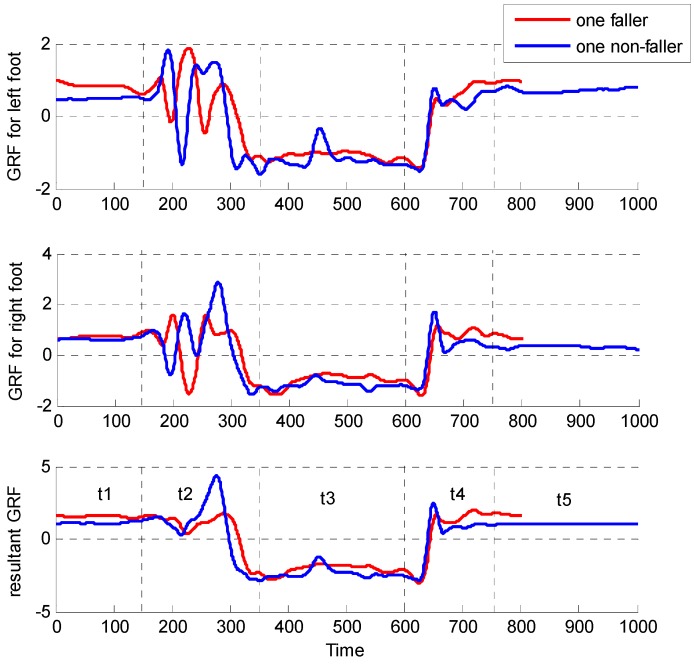
GRF on the force plate during STS movement. At the beginning of STS movement, the person keep on stand (t1). The time from stand to sit on t2, from sit to stand on t4. The curves for faller are smoother than the non-faller, with lower peak.

The second record is sit-to-stand (STS) [15]. Each subject was asked to stand on the MatScan^®^ system (TekScan, Boston, MA, USA), then sit down on an armless chair, and finally stand up. The vertical GRF time series data of both feet were recorded. As Figure 2 shows, the curves illustrated the ground reaction force for single fallers and non-fallers during the STS movement. It is indicated that there exist reaction force differences between the two groups. Old people could suffer falls due to the failure to perform STS movements. STS movements are typical daily life activities and are useful assessment of fall risk in older people.

### 2.3. Functional Scale Assessment

The first test is the Timed Up and Go (TUG) test [5]. The subjects stood up from the chair, walked 3 m, turned back, and then sat down on the chair. The time needed to execute this test evaluates the kinetic dependence of the subject.

The second test is the Dynamic Gait Index (DGI) test [6]. It can evaluate the lower extremity functions by several gait, stand up and balance tasks. This test is used to assess the balance ability and fall risk of the elderly. After all these tests, each participant receives a gait and balance report from the therapist. The data was saved to generate a database which could incrementally add new samples at runtime. These data were processed by the MATLAB software.

## 3. Feature Extraction and Sample Entropy

In the paper, Ground Reaction Forces (GRFs) were considered as effective factors for predictors of falls. In our study, the GRF data which were discussed, include L_ML_F, L_AP_F, L_SI_F, R_ML_F, R_AP_F, R_SI_F, L_V_F, R_V_F. Where, L_ML_F, L_AP_F, L_SI_F are the GRF for the left foot during walking in the medial-lateral, anterior-posterior and superior-inferior direction, respectively. R_ML_F, R_AP_F, R_SI_F are the GRF for the right foot during walking in the medial-lateral, anterior-posterior and superior-inferior direction, respectively. L_V_F, R_V_F represent the vertical GRF during STS for the left and right foot, respectively. The GRF features are listed in Table 1. To date, there is little literature reporting on the nonlinear characteristics of the GRF among fallers and non-fallers. Average and local features are always extracted to indicate multiple measurements of each subject, which could neglect some deterministic property and are easily contaminated with noise [16]. In fact, many old people have characteristic limb movements during walking or STS. Human limb movement is a complex dynamical system and indicate an irregular trend. In our study, the time series signals of these features were quantified by the sample entropy, which is a nonlinear measurement way introduced by Richman and Moorman [17] and less sensitive to data corrupted by noise [18]. Before calculating the sample entropy, GRF was normalized by the participant’s body weight. Each time series was standardized with zero mean and unit variance.

**Table 1:** The abbreviation of considered physical features.

No.	The Abbreviated Features	The Meaning of Features
1	L_ML_F	Medial-lateral GRF for left foot during walking
2	L_AP_F	Anterior-posterior GRF for left foot during walking
3	L_SI_F	Superior-inferior GRF for left foot during walking
4	R_ML_F	Medial-lateral GRF for right foot during walking
5	R-AP_F	Anterior-posterior GRF for right foot during walking
6	R_SI_F	Superior-inferior GRF for right foot during walking
7	L_V_F	Vertical GRF for left foot during STS
8	R_V_F	Vertical GRF for right foot during STS

Sample entropy is the negative natural logarithm of the conditional probability that two sequences that match for m points within a tolerance r remain similar at the next point, without allowing self-matches [17]. For each relevant feature, the sample entropy can be calculated after determining the constant values of m and r. Usually, the constant values of m is 1 or 2, the value r can take range from 0.1 to 0.25 [19]. For our data, we selected m = 2 and r = 0.25.

## 4. Feature Selection and Classification Method

K-nearest neighbor (KNN) rule [20] is one of the most popular and simplest nonparametric classification algorithms and can achieve good classification performances in many practical applications. It can learn from small sample size cases and acquire competitive performance compared with more modern methods such as support vector machines, neural networks and decision trees [21]. Moreover, the nearest neighbor classifiers are extremely sensitive to the considered features. That is, they are less effective when many features are irrelevant or noisy. For example, Langley and Iba [22] found that adding just a few irrelevant features could drastically change the nearest neighbor classifier’s output and reduce its accuracy. Using this instability, we are able to combine KNN with different selected features to generate a diverse set of classifiers and to compare different and hopefully classification performances for identifying non-fallers and fallers.

In statistical pattern recognition, it is well known that the performance of nonparametric classifiers is severely influenced by the existing outliers, particularly in small sample size situations [23]. To overcome the influences of the outliers on classification performance, we adopt the variation of the KNN-based approach.

The local mean-based k-nearest neighbor (LMKNN) rule [24] is one of those KNN-based variations. Firstly, LMKNN find k nearest neighbors for test sample x of each class, then calculate the local mean vector:

(1) $${\bar{x}}^{i}=\frac{1}{k}\sum_{l=1}^{k}x_{l}^{i}$$

where, x^i^_l_ is l-th training sample from class C_i._ Next, calculate the distance d(x, x^i^) between the test point and the local mean vector for each class. Finally, assign x into the class with the minimal distance d(x, x^i^).

The pseudo-nearest neighbor (PNN) rule [25] is another successful KNN-based classifier. PNN also find k nearest neighbors for test point x of each class, and give different weights to the k nearest neighbors according to their distances to x. The greater weight is assigned to the neighbor with the smaller distance. The weight w^i^_j_ of the j-th neighbor x^i^_j_ from the class C_i_ is defined as:

(2) $$w_{j}^{i}=\frac{1}{j},j=1,\cdots ,k$$

Next, calculate the weight distance sum for each class:

(3) $$d(x,x_{i}^{PNN})=w_{1}^{i}\times d\left(x,x_{1}^{i}\right)+w_{2}^{i}\times d\left(x,x_{2}^{i}\right)+\cdots +w_{k}^{i}\times d\left(x,x_{k}^{i}\right)$$

Finally, classify the test sample to into the class with the minimal weight distance sum.

The local mean pseudo-nearest neighbor classification (LMPNN) [26] is also an extension of the KNN rule. LMPNN first calculates the local mean vector x^i^_j_ of the first j nearest neighbors for a test sample x in each class:

(4) $${\bar{x}}_{j}^{i}=\frac{1}{j}\sum_{l=1}^{j}x_{l}^{i}$$

Then it nallocates different weights w^i^_j_ to k local mean vectors per class through Equation (2).

Next we calculate the distance sum between x and x^i^_j_ with w^i^_j_:

(5) $$d(x,{\bar{x}}^{i})=w_{1}^{i}\times d\left(x,{\bar{x}}_{1}^{i}\right)+w_{2}^{i}\times d\left(x,{\bar{x}}_{2}^{i}\right)+\cdots +w_{k}^{i}\times d\left(x,{\bar{x}}_{k}^{i}\right)$$

Finally the test sample x is assigned to the class with the minimum distance sum. Moreover, the Euclidean distance is used to identify the nearest neighbor.

The performance of the classification method is made by the leave-one-out cross validation (LOOCV) [27]. In this method, all but one sample undergo the learning step and the one remaining sample tests the learned algorithm. All samples should be retained and tested in turn, and the classification performance is obtained over the total number of samples in the dataset. Classification performance can be measured by the following criteria:

(6) $$Accuracy=\frac{TP+TN}{N}$$

(7) $$Sensitivity=\frac{TP}{TP+FN}$$

(8) $$Specificity=\frac{TN}{TN+FP}$$

TP means the number of fall samples which is correctly classified as fallers by the classifier. TN means the number of non-fall samples which is correctly classified as non-fallers. FP means the number of non-fall samples which are incorrectly classified as fallers. FN means the number of fall samples which are incorrectly classified as non-fallers. The total number of samples, the real number of fall samples, and the real number of non-fall samples, which was acquired in fact, are represented by N, N_1_, N_2_, respectively. There are some equations that relate these numbers: TP + FN = N_1_; FP + TN = N_2_; TP + FN + FP + TN = N.

Obviously, we could calculate the specificity through the accuracy and sensitivity. Sensitivity means the probability that fall samples are correctly classified as fallers. Sensitivity is the most important indicator of a fall detection algorithm, followed by the accuracy [28]. During feature selection, we should pay more attention to the sensitivity and accuracy.

The feature selection step, in classification algorithm design, means selecting a subset of features according to classification performances. A total of eight features were extracted from the 3 m walking tests and STS tests of each subject. Firstly, each of the features was considered independently, and then any possible combinations of these features were discussed. The total number of different combinations of eight features which could be studied in this paper, is 255. Then each subset is separately trained and tested by using the KNN-based nearest neighbor classifiers, and the subset with the greatest classification performance is chosen.

## 5. Statistical Analysis

Here, we further employ two non-parametric statistical tests—Wilcoxon rank sum test and Spearman Correlation analysis—to verify the performance of those selected features.

Because of the asymmetrical comparison of two groups, Wilcoxon rank sum test is applied to investigate whether there are significance differences between the sample entropies of the features. To investigate the degree of relationship between features, Spearman Correlation analysis is the appropriate method because it does not need to need any assumptions about the distribution of the data.

## 6. Results

### 6.1. Characteristics of the Participants

No differences existed in age and body weight among the fallers and non-fallers according to the t-test. In addition, there were no significant differences between fallers and non-fallers in gender, number of medications and diseases via the chi-square test. The characteristics of both groups of participants are listed in Table 2.

**Table 2:** Characteristics of the participants in both groups. Values are shown as MEAN ± SD (standard deviation) in two groups; *p*-values are based on t-tests comparing continuous data or chi-square tests comparing categorical data.

Characteristic	Faller (n = 23)	Non-Faller (n = 15)	*p*-Value
Age (years)	72.29 ± 4.98; 65–84	69.93 ± 4.51; 65–78	0.12
Gender (%men)	42.85%	45.83%	0.99
Weight (kg)	65.92 ± 10.17	58.33 ± 18.18	0.16
Number of medications	1.45 ± 0.97	1.5 ± 1.09	0.91
Number of diseases	1.08 ± 1.34	0.86 ± 1.1	0.57

### 6.2. The Functional Scale Assessment of the Two Groups

The functional scales assessments included the TUG and DGI tests. Significant differences were indicated among the two groups. The fallers had higher total scores than the non-fallers. According to the falling experience and the scores obtained at the functional scale tests (TUG and DGI tests) mentioned above, these evaluations are used as the fall risk standard. All participants were categorized as twenty-three fallers and fifteen non-fallers, but there was an exception. Participant 36 belonged to the not at risk group, but his scale test score was so high that it encouraged us to classify him in the fallers group.

### 6.3. Classification Results

As mentioned above, eight features quantified by sample entropy were selected to calculate the accuracy, sensitivity and specificity based on the LMPNN, PNN, and LMKNN algorithms, respectively. The total number of combinations of eight features was 255. When selecting the optimal feature set, we firstly consider the value of sensitivity, followed by the value of accuracy. Table 3 shows the optimal features and relevant classification rate for the three classification algorithms. The first column shows the classification algorithm. The second one showed the final subset of all the features for the relevant classification. The third, fourth, and fifth columns gave the accuracy, sensitivity and specificity rate for these feature sets. Table 3 also shows that the LMPNN outperform the other algorithms with 100% of accuracy, 100% of sensitivity and 100% of specificity.

**Figure 3:**
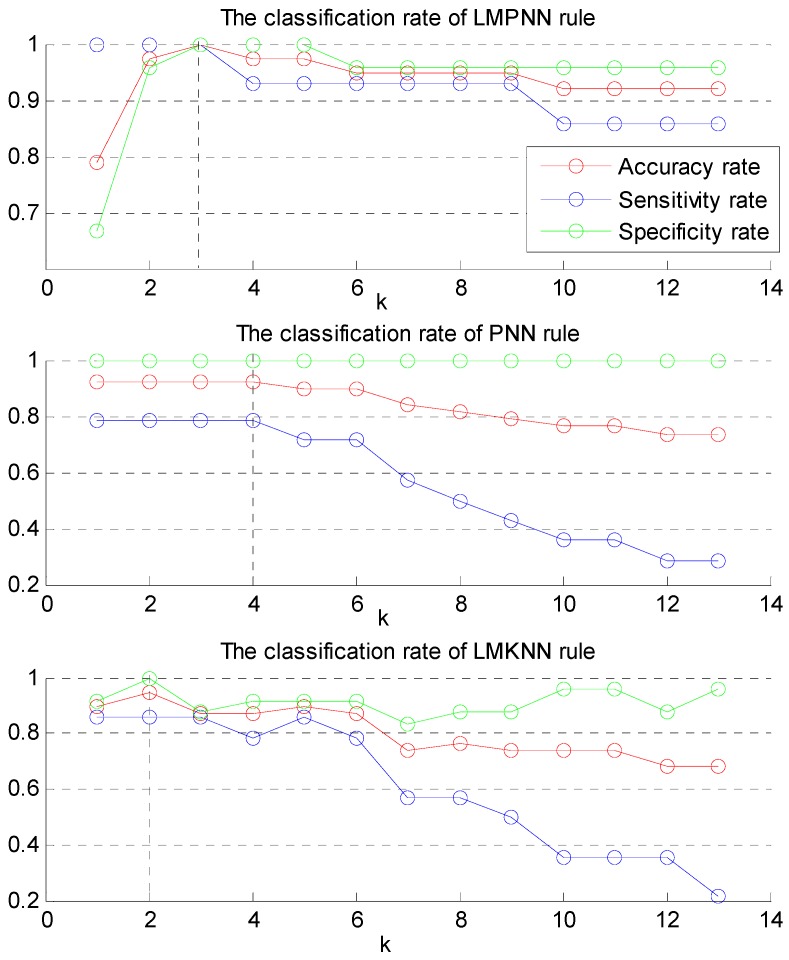
The classification rates of LMPNN, PNN, and LMKNN on real data via different k nearest neighbor methods.

Figure 3 shows the classification rates of LMPNN, PNN, and LMKNN on real data via different k nearest neighbors. The values of k influenced the classification rate. It was observed that the classification rate reached the maximum when k was equal to 3, 4, and 2 for LMPNN, PNN and LMKNN, respectively.

**Table 3:** The selected features and relevant classification performance for the three classification algorithms.

Algorithm	Select Features	Accuracy Rate	Sensitivity Rate	Specificity Rate
LMPNN	L_SI_F, R_ML_F, R_AP_F, L_V_F (k = 3)	100%	100%	100%
PNN	L_SI_F, R_ML_F, R_AP_F, L_V_F, R_V_F (k = 1/2/3/4)	92.11%	78.57%	100%
LMKNN	L_SI_F, R_ML_F, R_AP_F, L_V_F (k = 2)	94.74%	85.71%	100%

The optimal value of accuracy, sensitivity and specificity rate exceed the median sensitivity and specificity scores [3], which were 85% and 78% for the functional assessment tools, respectively. The optimal feature subsets were {L_SI_F, R_ML_F, R_AP_F, L_V_F}, {L_SI_F, R_ML_F, R_AP_F, L_V_F, R_V_F}, {L_SI_F, R_ML_F, R_AP_F, L_V_F} for LMPNN, PNN and LMKNN, respectively. In conclusion, the selected features included L_SI_F, R_ML_F, R_AP_F, L_V_F.

### 6.4. Comparisons and Relationships of Sample Entropy for Features

With regard to the corresponding features measured by sample entropy, the p values of the Wilcoxon rank sum test was given as 0.2092, 0.1341, 0.0586, 0.09879, 0.0329, 0.3254, 0.0097, 0.0081, respectively, as shown in Table 4. Significance differences exist if *p*-value < 0.1. There were significant differences between the fallers and non-fallers in L_SI_F, R_ML_F, R_AP_F, L_V_F, R_V_F.

Moreover, the following scores could be used to evaluate the degree of correlation [29]:

1. |r| ≥ 0.50: high correlation;
2. 0.30 ≤ |r| ≥ 0.49: moderate correlation;
3. 0.10 ≤ |r| ≥ 0.29: weak correlation.

In Table 5, the Spearman correlation coefficients and *p*-values are listed for selected features. There is a moderate correlation between L_SI_F and R_AP_F (r = 0.361, *p* = 0.026); L_SI_F and R_ML_F are moderately correlated (r = 0.493, *p* = 0.002); L_V_F is also highly correlated with R_V_F (r = 0.547, *p* = 0.000).

**Table 4:** The comparison of fallers with non-fallers using the Wilcoxon rank sum test on sample entropies concerning eight features. Values are shown as MEAN ± SD (Standard deviation) in two groups; significant results are indicated with an asterisk (*).

The Abbreviated Features	Faller	Non-Faller	*p*-Value
L_ML_F	0.5586 ± 0.1389	0.6246 ± 0.1858	0.2092
L_AP_F	0.4496 ± 0.0915	0.4835 ± 0.0421	0.1341
L_SI_F	0.2574 ± 0.1655	0.2819 ± 0.0690	0.0586 *
R_ML_F	0.5700 ± 0.1172	0.5826 ± 0.1963	0.09879 *
R_AP_F	0.4661 ± 0.0986	0.5116 ± 0.0574	0.0329 *
R_SI_F	0.2996 ± 0.1485	0.3187 ± 0.1144	0.3254
L_V_F	0.0852 ± 0.0297	0.1110 ± 0.0313	0.0097 *
R_V_F	0.1003 ± 0.0402	0.1339 ± 0.0340	0.0081 *

**Table 5:** Spearman correlation coefficients among the select features. Significant results are indicated with an asterisk (*).

		L_SI_F	R_ML_F	R_AP_F	L_V_F	R_V_F
L_SI_F	r	1	0.493 *	0.361 *	0.165	0.315
	*p*-value	--	0.002	0.026	0.323	0.054
R_ML_F	r	0.493 *	1	0.121	0.297	0.188
	*p*-value	0.002	--	0.469	0.070	0.258
R_AP_F	r	0.361 *	0.121	1	0.188	0.205
	*p*-value	0.026	0.469	--	0.258	0.217
L_V_F	r	0.165	0.297	0.188	1	0.547 *
	*p*-value	0.323	0.070	0.258	--	0.000
R_V_F	r	0.315	0.188	0.205	0.547 *	1
	*p*-value	0.054	0.258	0.217	0.000	--

## 7. Discussion

The classification results indicated that the superior-inferior GRF for left foot during walking, anterior-posterior and medial-lateral GRF for right foot during walking, and vertical GRF for left foot during STS could predict previous falling events and be useful in fall risk assessment. Significant difference and the Spearman correlation coefficients were compatible with the classification results.

We successfully classified the elderly into two groups with great classification performance. However, such performance with 100% results may not be maintained when the algorithm is applied to other participants. Although some academic works [21,30] with high sensitivity and specificity exist, the performance of the algorithms in these studies degrades when implemented in the real world under realistic conditions or with new users.

To date, only a few studies have suggested that force platform-based balance measurements can be used as predictors of falls among elderly populations [11]. Our study adds knowledge in this research field, by the use of the force platform method which can collect valid ground reaction force data. These data, which are quantified by sample entropy, are useful in the identification of people at risk for falls.

The reason why we enter the 3-axis GRFs during walking into the fall classification system is because these features are typically used to identify normal and pathological human gaits, and could be used as indicators of falling. Another study has also described the relationships between the 3-axis GRFs during walking and fall prevention [14]. On the one hand, the walking ability of the elderly is expressed in walking speed and stability. The anterior-posterior ground reaction force is the driving forces which is considered to be a factor affecting the walking speed in elderly people. Nilsson and Thorstensson [31] have reported that walking speed can increase, when the anterior-posterior ground reaction force increases. As muscle strength and balance decrease, there is a difference between the fallers and non-fallers in the horizontal component of the ground reaction force. Moreover, the superior-inferior ground force reaction reflects the vertical force which is associated with the stability of the subject. When the vertical force is higher (lower) than the subject’s weight, the subject moves upward (downward). Usually, the vertical force of people who have lower limb muscle injuries and a history of falling down is smoother than that of normal elderly people. On the other hand, asymmetry exists in feet pressure between the left and right feet during gait [32]. Several previous reports have also indicated that the left limb was found to be responsible for support, and the right limb associated with the propulsion [33], which is compatible with our study.

In addition, STS movement is one of the fundamental daily living activities. The STS test could be a useful and practical test, which reflects the lower limb function of the old people and can appropriately predict falling accidents. Falls often occur with high probability when losing stability and balance during the STS movement [34]. The vertical GRF for feet during STS are associated with strength of the knee extensor or flexor muscle, the ankle flexor muscles and with joint motion, balance [35], which is useful for measuring lower-limb muscle strength and power in the elderly population. A previous study demonstrated that the maximal lower muscle power which was calculated by the vertical GRF during STS was a significant independent parameter that discriminated whether elderly people had falling experience or not [36]. Yamada *et al.* [15] have also reported that there was a high correlation between GRF parameters during STS movement and falling risk of the elderly. Although the subjects’ characteristics extracted from ground reaction force were different in the previous study, the STS movement has been considered useful to predict the occurrence of falling induced by physical lower limb function decreases. Cheng *et al.* [36] also found that there is no significant difference in the maximal vertical GRF between fallers and non-fallers. However, it has been found that sample entropy of vertical ground reaction forces for the feet during STS were different in our study. This may indicate that, compared with some of the linear methods, there are certain advantages in providing information using sample entropy measurement.

These static and dynamic ground reaction forces could play a crucial role in predicting fall risk. Whether the optimal features quantified by sample entropy are associated with falling accidents has not been previously researched. The present study clearly indicated that these measured features could be predictors of previous falling events.

In this study, each subject was instructed to walk and sit-to-stand on several times for the sake of collecting enough information to perform the analysis. A previous study indicated that repeating sit-to-stand five times could predict further falling and disability risks in daily activities [37]. However, we only considered two times walking and one time STS tests. In future studies, the optimal test time should be determined for statistical processing. There is a limitation in our method, which refers to the appearance of a dependence of the nearest neighbor classification on the sample density. Indeed, the classification performance will be great when the sample density of each category is great and sensible. We will add new samples at runtime to verify the superiority of the selected features set which could be a predictor of past falling events and generate an objective fall risk assessment system.

## 8. Conclusions

In this study, we aimed to determine whether objective measures of physical function could predict subsequent fall risk in older persons. From the present study, the following conclusions can be drawn:

1. For the sake of quantifying time series signals of GRF features, the sample entropy was calculated when the constant values of m and r were 2, 0.25, respectively.
2. We successfully classified the elderly into two groups: at risk and not at risk using three KNN-based classifiers: local mean-based k-nearest neighbor (LMKNN), pseudo-nearest neighbor (PNN) and local mean pseudo-nearest neighbor (LMPNN) classification. We compare the performance of the classifiers, and achieve the best results with LMPNN, with sensitivity, specificity and accuracy is 100%, 100%, 100%, respectively.
3. The statistical characteristics of the feature subset differed significantly between the fallers and non-fallers. Statistical differences were found for the following features: sample entropies of superior-inferior GRF for left foot during walking; sample entropies of medial-lateral and anterior-posterior GRF for right foot during walking; sample entropies of vertical GRF for double feet during STS.
4. The final and selected features included the superior-inferior GRF for left foot during walking, medial-lateral and anterior-posterior GRF for right foot during walking, and the vertical GRF for left foot during STS.

The results can be potentially used for evaluation of lower limb extremity function, automatic classification for fall risk.
